# Automated DNA-Encoded Library Synthesis on Magnetic Anion Exchange Resin

**DOI:** 10.64898/2026.10.21.760614

**Published:** 2026-10-22

**Authors:** Valerie Cavett, Santino L. DeGirolamo, Erika M. Cerna Arroyo, Robert Damoiseaux, Brian M. Paegel

## Abstract

DNA-encoded libraries (DELs) access vast chemical spaces for drug discovery, but split-and-pool DEL synthesis remains the sole and very manual portion of the DEL workflow. Here, we automate DEL construction using diethylaminoethyl-functionalized weak anion exchange (WAX) magnetic resin and a KingFisher Apex particle-handling instrument. DNA encoding occurs in solution, followed by selective immobilization of DNA conjugates under low-salt conditions that exclude > 95% of unused encoding oligonucleotides. On-DNA chemistry then proceeds using the WAX resin-adsorbed “WAX-ON” DNA substrate in neat organic solvent. The platform supports amide formation, reductive amination, copper-catalyzed azide–alkyne cycloaddition, and Fmoc removal. Automated bead transfer enables iterative reaction and washing cycles. We synthesized two-cycle 9,216-member DELs from 96 Fmoc-protected amino acids and 96 carboxylic acids. Control selections reproducibly enriched the carbonic anhydrase ligand sulfamoylbenzoic acid. Magnetic WAX-ON DEL synthesis is compatible with diverse chemistries and uses the same instrumentation to automate DEL construction and affinity screening.

## Full Text Availability

The license terms selected by the author(s) for this preprint version do not permit archiving in PMC. The full text is available from the preprint server.

